# Convergence is Only Skin Deep: Craniofacial Evolution in Electric Fishes from South America and Africa (Apteronotidae and Mormyridae)

**DOI:** 10.1093/iob/obac022

**Published:** 2022-08-13

**Authors:** Kassandra L Ford, Rose Peterson, Maxwell Bernt, James S Albert

**Affiliations:** Institute of Ecology and Evolution, Universität Bern, Switzerland; Department of Fish Ecology and Evolution, Eawag Swiss Federal Institute of Aquatic Science and Technology, Switzerland; Department of Biology, University of Louisiana at Lafayette, USA; Department of Biological Sciences, George Washington University, USA; Department of Biology, University of Louisiana at Lafayette, USA; Department of Ichthyology, American Museum of Natural History, USA; Department of Biology, University of Louisiana at Lafayette, USA

## Abstract

Apteronotidae and Mormyridae are species-rich clades of weakly electric fishes from Neotropical and Afrotropical freshwaters, respectively, known for their high morphological disparity and often regarded as a classic example of convergent evolution. Here, we use CT-imaging and 3D geometric morphometrics to quantify disparity in craniofacial morphologies, and to test the hypothesis of convergent skull-shape evolution in a phylogenetic context. For this study, we examined 391 specimens representing 78 species of Apteronotidae and Mormyridae including 30 of 37 (81%) of all valid genera with the goal to sample most of the craniofacial disparity known in these clades. We found no overlap between Apteronotidae and Mormyridae in the skull-shape morphospace using PCA and a common landmark scheme, and therefore no instances of complete phenotypic convergence. Instead, we found multiple potential instances of incomplete convergence, and at least one parallel shift among electric fish clades. The greatest components of shape variance in both families are the same as observed for most vertebrate clades: *heterocephaly* (i.e., opposite changes in relative sizes of the snout and braincase regions of the skull), and *heterorhynchy* (i.e., dorsoventral changes in relative snout flexion and mouth position). Mormyrid species examined here exhibit less craniofacial disparity than do apteronotids, potentially due to constraints associated with a larger brain size, ecological constraints related to food-type availability. Patterns of craniofacial evolution in these two clades depict a complex story of phenotypic divergence and convergence in which certain superficial similarities of external morphology obscure deeper osteological and presumably developmental differences of skull form and function. Among apteronotid and mormyrid electric fishes, craniofacial convergence is only skin deep.

## Synopsis

Les Apteronitidae et Mormyridae sont des clades riches en espèces de poissons faiblement électriques que l'on trouve respectivement dans les eaux douces des zones Néo-tropicale et Afro-tropicale. Ils sont connus pour leurs disparités morphologiques et sont souvent utilisés comme un exemple de convergence évolutive. Dans cette étude, nous avons utilisé l'imagerie TDM et la morphométrie géométrique 3D pour quantifier les morphologies cranio-faciales et tester les hypothèses de convergence sur l’évolution de la forme du squelette dans un contexte phylogénétique. Nous avons examiné 391 spécimens représentant un total de 78 espèces d'Apteronitidae et de Mormyridae et incluant 30 des 37 (81%) genres représentés dans ces clades avec l'objectif d’échantillonner la majorité des disparités cranio-faciales connues. Les résultats de l'ACP et de l'analyse basée sur les “landmarks” n'ont pas révélé de chevauchement dans la forme du crâne entre les deux clades. Cependant, nous avons trouvé de multiples indices en faveur d'une convergence incomplète, et au moins un évènement de déplacement parallèle entre ces clades de poissons électriques.

Les facteurs contribuant le plus à la variance dans la forme des deux familles sont les mêmes que ceux observés chez la plupart des vertébrés : l'hétéro-céphalie (variation opposée dans la taille relative du museau et de la boite crânienne) et l'hétéro-rhynchie (les changements dorso-ventraux dans la courbure du museau par rapport à la position de la bouche). Les espèces de Mormyridae examinées dans cette étude présentent moins de disparités cranio-faciales que les Apteronitidae. Cela peut être dû à des contraintes associées à un cerveau de plus grande taille ou encore à des contraintes écologiques liées à la disponibilité des différentes ressources.

Les patrons d’évolution cranio-faciale présents dans ces deux clades démontrent une histoire de divergence phénotypique et de convergence complexe dans laquelle certaines similarités dans la morphologie externe rendent difficilement observable de plus profondes différences ostéologiques et probablement développementales dans la forme et la fonction du crâne. Au sein des poissons électriques appartenant aux clades des Apteronitidae et Mormyridae, il semble que la convergence cranio-faciale soit seulement superficielle.

## German Zusammenfassung

Apteronotidae und Mormyridae sind artenreiche Familien von schwach elektrischen Fischen aus Neotropischen und Afrotropischen Flüssen und Seen, welche bekannt für ihre hohe morphologische Disparität sind und oft als klassisches Beispiel von konvergenter Evolution herangezogen werden. Hier verwenden wir Computer-Tomographische Bild gebende Verfahren (CT-Scans) und 3D geometrische Morphometrie, um die Disparität in kraniofazialer Morphologie zu quantifizieren und die Hypothese von konvergenter Evolution bezüglich Schädel-Form in einem phylogenetischen Kontext zu testen. Für diese Studie untersuchten wir 391 Individuen aus 78 Arten aus den beiden Familien Apteroniotidae und Mormyridae, wobei Arten 30 von 37 gültigen Gattungen (81%) benutzt wurden, mit dem Ziel, die grössten kraniofazialen Disparitäten in diesen Gruppen abzudecken. Anhand einer PCA basierend auf einem gemeinsamen Markierungs-Schema (landmarking) fanden wir keine Überlappung zwischen Apteronotidae und Mormyridae in Schädel-Form Morphospace und daher auch keine Beispiele von kompletter phänotypischer Konvergenz. Stattdessen fanden wir mehrere potenzielle Fälle von inkompletter Konvergenz und mindestens einen Fall von paralleler Verschiebung zwischen diesen Gruppen von elektrischen Fischen. Die grössten Komponenten in der Varianz der Form bzw. Gestalt in beiden Familien sind dieselben, die in den meisten Wirbeltier-Gruppen beobachtet werden: *heterocephaly* (d.h. entgegengesetzte Veränderungen in relativer Grösse der Schnauzen- und Gehirnschädel-Region des Schädels) und *heterorhynchy* (d.h. dorsoventrale Veränderungen in relativer Schnauzen-Krümmung und Maul-Position). Arten der Familie Mormyridae zeigen weniger kraniofaziale Disparität als Arten der Familie Apteronotidae, möglicherweise wegen der grösseren Gehirn-Grösse und ökologischer Einschränkungen in Zusammenhang mit der Verfügbarkeit von verschiedenen Nahrungsmitteltypen. Die Muster von kraniofazialer Evolution in diesen beiden Gruppen zeichnen eine komplexe Geschichte von phänotypischer Divergenz und Konvergenz in welcher gewisse oberflächhliche Ähnlichkeiten der externen Morphologie tieferliegende osteologische und vermutlich entwicklungsbiologische Unterschiede in der Schädelform und –funktion überdecken. Kraniofaziale Konvergenz geht bei den Apteronotiden und den Mormyiden nicht unter die Haut.

## Portuguese Resumo

Apteronotidae e Mormyridae são clados ricos em espécies de peixes elétricos de fraca descarga de água doce Neotropical e Afrotropical, respectivamente, conhecidos por sua alta disparidade morfológica e frequentemente considerado como um exemplo clássico de convergência de evolução. Nós usamos aqui, imagens de tomografia computadorizada e morfometria geométrica 3D para quantificar a disparidade morfológica craniofacial e testar a hipótese de convergência evolutiva da forma do crânio em um contexto filogenético. Para este estudo, nós examinamos 391 espécimes representando 78 espécies de Apteronotidae e Mormyridae, incluindo 30 dos 37 (81%) gêneros válidos com o objetivo de amostrar a maior parte da disparidade craniofacial conhecida nestes clados. Nós não encontramos sobreposição no morfoespaço da forma do crânio entre Apteronotidae e Mormyridae usando PCA e um esquema comum de pontos de referência, e portanto, nenhum caso de convergência evolutiva completa. Entretanto, nós encontramos múltiplos potenciais casos de convergência incompleta, e ao menos uma mudança paralela entre clados de peixes elétrico. Os maiores componentes da variância da forma nas duas famílias são os mesmos observados para a maioria dos clados dos vertebrados:: *heterocephaly* (i.e., mudanças opostas em tamanhos relativos do focinho e regiões da caixa craniana), e *heterorhynchy* (i.e., mudanças dorsoventrais na flexão relativa do focinho e posição da boca). As espécies de mormyrídeos aqui examinadas, exibem menos disparidade craniofacial que os apteronotídeos, potencialmente devido à limitações associadas com o tamanho maior do cérebro e restrições ecológicas relacionada a disponibilidade de tipo de alimento. Padrões de evolução craniofacial nestes dois clados ilustra uma história complexa de divergência fenotípica e convergência em que certas similaridades superficiais da morfologia externa obscurece as diferenças mais profundas osteológicas e presumivelmente de desenvolvimento da forma e função do crânio. Entre os peixes elétricos apteronotídeos e mormyrídeos, a convergência craniofacial é somente aparente.

## Spanish Resumen

Apteronotidae y Mormyridae son clados ricos en especies de peces débilmente eléctricos de aguas dulces Neotropicales y Afrotropicales, respectivamente, conocidos por su alta disparidad morfológica y, a menudo, considerados como un ejemplo clásico de evolución convergente. Aquí usamos imágenes de tomografía computarizada y morfometría geométrica 3D para cuantificar la disparidad en las morfologías craneofaciales y para probar la hipótesis de la evolución convergente de la forma del cráneo en un contexto filogenético. Para este estudio, examinamos 391 especímenes que representan 78 especies de Apteronotidae y Mormyridae, incluidas 30 de 37 (81%) de todos los géneros válidos, con el objetivo de muestrear la mayor parte de la disparidad craneofacial conocida en estos clados. No encontramos superposición entre Apteronotidae y Mormyridae en el morfoespacio en forma de cráneo utilizando PCA y un esquema de referencia común y, por lo tanto, no hay instancias de convergencia fenotípica completa. En cambio, encontramos múltiples instancias potenciales de convergencia incompleta y al menos un cambio paralelo entre los clados de peces eléctricos. Los mayores componentes de la variación de la forma en ambas familias son los mismos que se observan en la mayoría de los clados de vertebrados: *heterocephaly* (es decir, cambios opuestos en los tamaños relativos del hocico y las regiones de la caja craneal del cráneo) y *heterorhynchy* (es decir, cambios dorsoventrales en la flexión relativa del hocico y posición de la boca). Las especies de mormíridos examinadas aquí exhiben menos disparidad craneofacial que los apteronótidos, posiblemente debido a limitaciones asociadas con un tamaño cerebral más grande, limitaciones ecológicas relacionadas con la disponibilidad de tipo de alimento. Los patrones de evolución craneofacial en estos dos clados representan una historia compleja de divergencia y convergencia fenotípica en la que ciertas similitudes superficiales de la morfología externa oscurecen diferencias osteológicas más profundas y presumiblemente de desarrollo de la forma y función del cráneo. Entre los peces eléctricos apteronótidos y mormíridos, la convergencia craneofacial es sólo superficial.

## Introduction

**Fig. 1:**
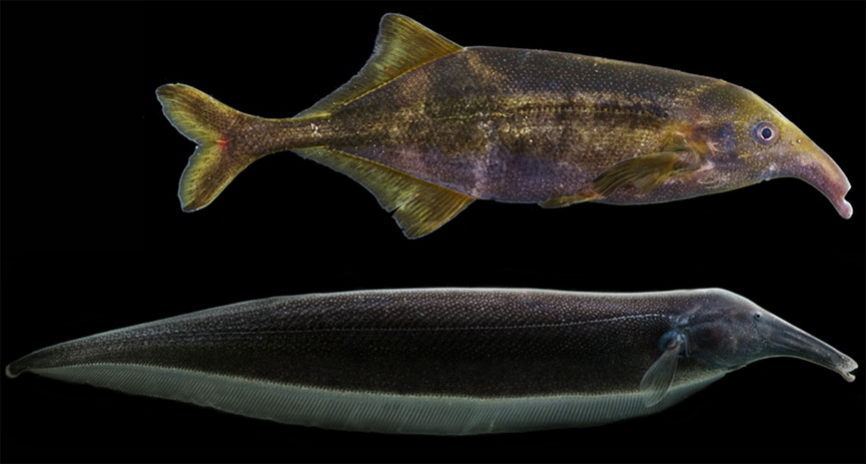
An apteronotid and mormyrid species with a full-body view. Full-body images of the mormyrid *Campylomormyrus elephas* (top) and apteronotid *Sternarchorhynchus cramptoni* (bottom) showing phenotypic similarities and differences. The body shapes differ in that mormyrids possess paired pectoral and pelvic fins as well as well-developed median anal, dorsal, and caudal fins, while apteronotids possess an elongate anal fin, a reduced caudal fin, and lack pelvic and dorsal fins. Photo credits: John P. Sullivan (Mormyridae) and Danté Fenolio (Apteronotidae). The authors and photographers request the images in this figure not be downloaded for separate use.

Convergent evolution, referring to the independent origins of similar traits in distantly related species, is widely considered resulting from selection for phenotypes that solve similar functional, physiological, or ecological problems (Revell et al. 2007; Losos 2011; Stayton 2015; Ord and Summers 2015; Sackton and Clark 2019; Grossnickle et al. 2020). Under the umbrella idea of convergence, traits can vary in the degree of structural or functional similarities, and also in the covariances of similarities among levels in the hierarchy of organismal design (Striedter and Northcutt 1991). These levels, including genetics, development, morphology, and function, can appear as convergent or divergent across species, and there is not always a one-to-one connection between them (different morphologies may result in the same behavior, such as flight in birds and bats) (Striedter and Northcutt 1991). Even at the morphological level, we cannot assume that internal morphologies are completely homologous across taxa, even if external morphologies appear similar (Shubin et al. 2009). Morphologists have used morphotypes (qualitative external shape categories) as heuristics for understanding the ecology and evolution of organismal trait evolution (Cresko and Baker 1996; Berrebi and Valiushok 1998; Dimmick et al. 2001; Meier et al. 2017; Meier et al. 2019; McGee et al. 2020). They assign species to distinct phenotypic categories to make inferences about ecology and habitat use (Cresko and Baker 1996; Berrebi and Valiushok 1998; Dimmick et al. 2001; Meier et al. 2017; Meier et al. 2019; McGee et al. 2020). While morphotypes are useful for identifying possible instances of convergence, new phylogenetic comparative methods and ways to quantify shape are better suited for analyzing convergent evolution. In this paper, we utilize these cutting-edge methods to study convergent evolution in apteronotid and mormyrid weakly electric fishes (Fig. 1).

In phylogenetic comparative methods, researchers have put forward definitions to identify different types of convergence and quantify shape differences among taxa. Complete convergence can be said to occur when species or higher taxa from phylogenetically distant clades exhibit considerable or total overlap in their phenotypes (Losos 2011; Meachen-Samuels 2012), as represented, for example, in a multivariate shape-space where taxa are connected by lines depicting their phylogenetic interrelationships; i.e., a phylomorphospace (Sidlauskas 2008). By the same token, incomplete convergence occurs when taxa in a phylomorphospace are closer together than were their ancestors, although still occupying distinct portions of the phylomorphospace (see Fig 3. in Stayton 2006). Another possibility is parallel evolution (coined “parallel shifts”), in which different lineages undergo similar morphological changes represented as parallel lines in a phylomorphospace, often thought to arise from changes in similar underlying genetic or developmental factors affecting the production of phenotypic variation (Simpson 1961; Schluter et al. 2004; Arendt and Reznick 2008; Bolnick et al. 2018). The development of new phylogenetic comparative methods has increased the reliability and analytical tractability of quantifying these different modes of phenotypic evolution using statistical models (Stayton 2015; Grossnickle et al. 2020). These methods also permit us to differentiate between different types of convergence using morphological and morphometric data, and multivariate statistical and comparative phylogenetic analyses.

Our study aims to examine internal morphological characters of two distantly-related groups of electric fishes, Gymnotiformes and Mormyridae, for patterns of convergent evolution. Gymnotiformes are a clade of weakly electric fishes from the humid Neotropics containing approximately 262 valid species (Albert 2001; 2003; Albert and Crampton 2006; 2009; Evans et al. 2017; Bernt et al. 2018; 2019; Ivanyisky and Albert 2014). In this group, the family Apteronotidae (with 99 valid species) includes a high proportion of total morphological disparity observed in Gymnotiformes as a whole, particularly in terms of head shape and craniofacial phenotypes (Albert 2001; Evans et al. 2017; Ford et al. 2022). Salient diagnostic characters of Apteronotidae include: presence of a small caudal fin with segmented fin rays, an elongate fleshy electroreceptor organ located on the dorsal body margin, and a neurogenic electric organ that generates a high-frequency wave-type electric signal (Bennett 1965; Albert 2001; Albert and Crampton 2005; Bernt et al. 2019). Ford additional diagnostic osteological traits see Albert (2001). Apteronotid species inhabit most aquatic habitats of lowland tropical South America, and are most diverse in deep (> 5 m) and swiftly flowing river channels of large Amazonian rivers. The high disparity in head, snout, and mouth shape in deep channel apteronotids is accompanied by high trophic diversity, and these traits are hypothesized to represent adaptations to utilize different habitats and trophic resources (Marrero and Winemiller 1993; Winemiller and Adite 1997; Albert and Crampton 2005; Albert and Reis 2011; Evans et al. 2019).

Mormyrid fishes are another clade of weakly electric fishes from the Afrotropics with approximately 227 valid species (van der Bank and Kramer 1996; Lavoué et al. 2004; Kramer 2013; Lamanna et al. 2016). As with apteronotids, mormyrids exhibit high disparity of head shape, craniofacial phenotypes, and electric signal waveforms (Ford et al. in review; van der Bank and Kramer 1996; Lavoué et al. 2004; Kramer 2013; Lamanna et al. 2016). Mormyrids have a relatively large brain as compared to their body size, hypothesized to be connected to cognitive functions such as environmental mapping and signal recognition (Nilsson 1996; von der Emde and Bleckmann 1998; Butler and Hodos 2005; Striedter 2005; Sukhum et al. 2016). Salient diagnostic characters of Mormyridae include: non-protrusible mouths; cycloid scales covering the body; small pores in the skin covering the body and head; posterior dorsal and anal fins; and a deeply forked caudal fin with rounded lobes (Kramer 1994; Sullivan and Hopkins 2005; Kramer 2013; Hilton and Lavoue 2018; Amen et al. 2020; Mulelenu et al. 2020). Additional diagnostic osteological traits are provided by Hilton (2003). Mormyrids inhabit many habitats across continental Africa, including small streams, fast-moving rivers, and swamps (Chapman et al. 2002; Montchowui et al. 2007; Lavoué et al. 2012; Jackson et al. 2013; Adjibade et al. 2020). Mormyrids exhibit moderate levels of dietary diversity, hypothesized to have contributed to the disparity of head and mouth phenotypes observed in this family (Okedi 1971; Fawole 2002; Arnegard and Carlson 2005; N'da et al. 2014).

**Fig. 2:**
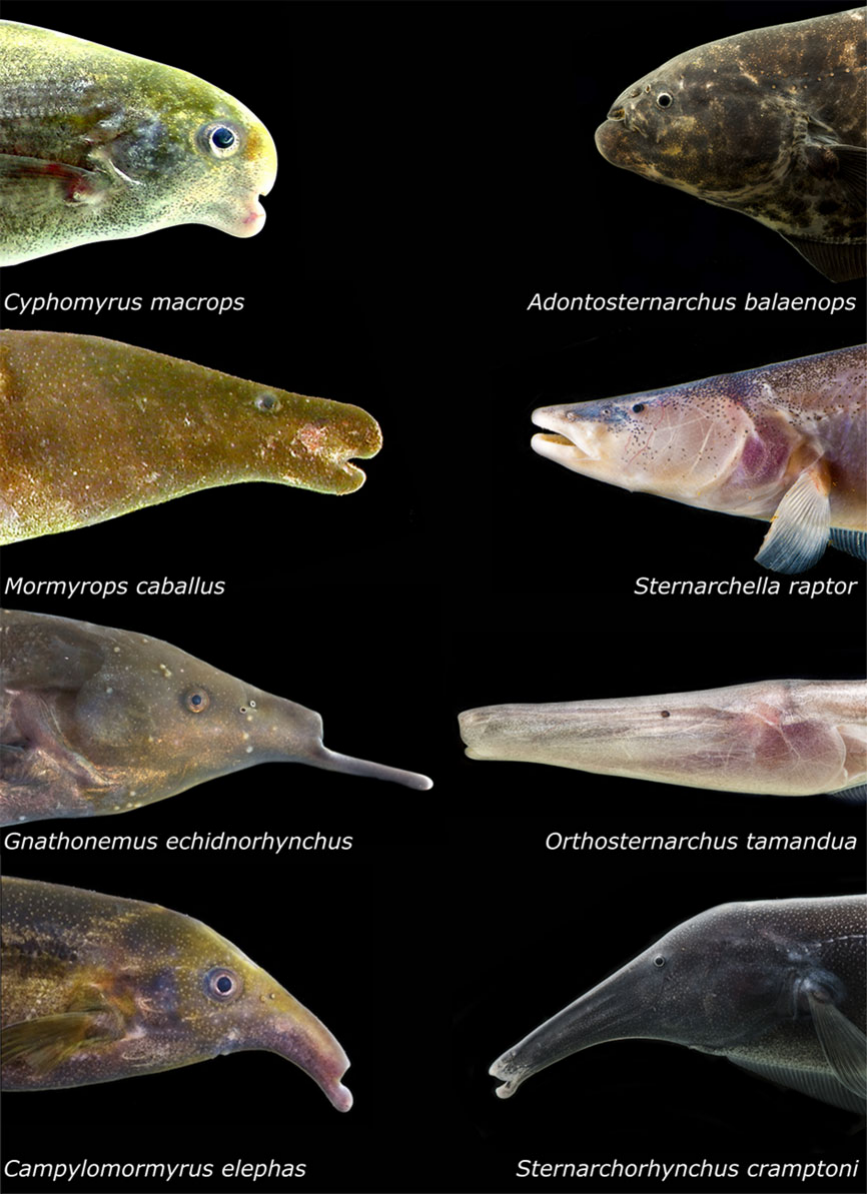
Headshots of four apteronotid and four mormyrid species. Four species each of Mormyridae (left) and Apteronotidae (right) illustrating similarities and differences in external head shape. Each family includes species with a short snout (top row), intermediate-length snout (second row), long snout with large mouth (third row), and long snout with small mouth (bottom row). Photo credits: John P. Sullivan (Mormyridae) and Danté Fenolio (Apteronotidae). Note smaller eye size in Apteronotidae. The authors and photographers request the images in this figure not be downloaded for separate use.

**Fig. 3:**
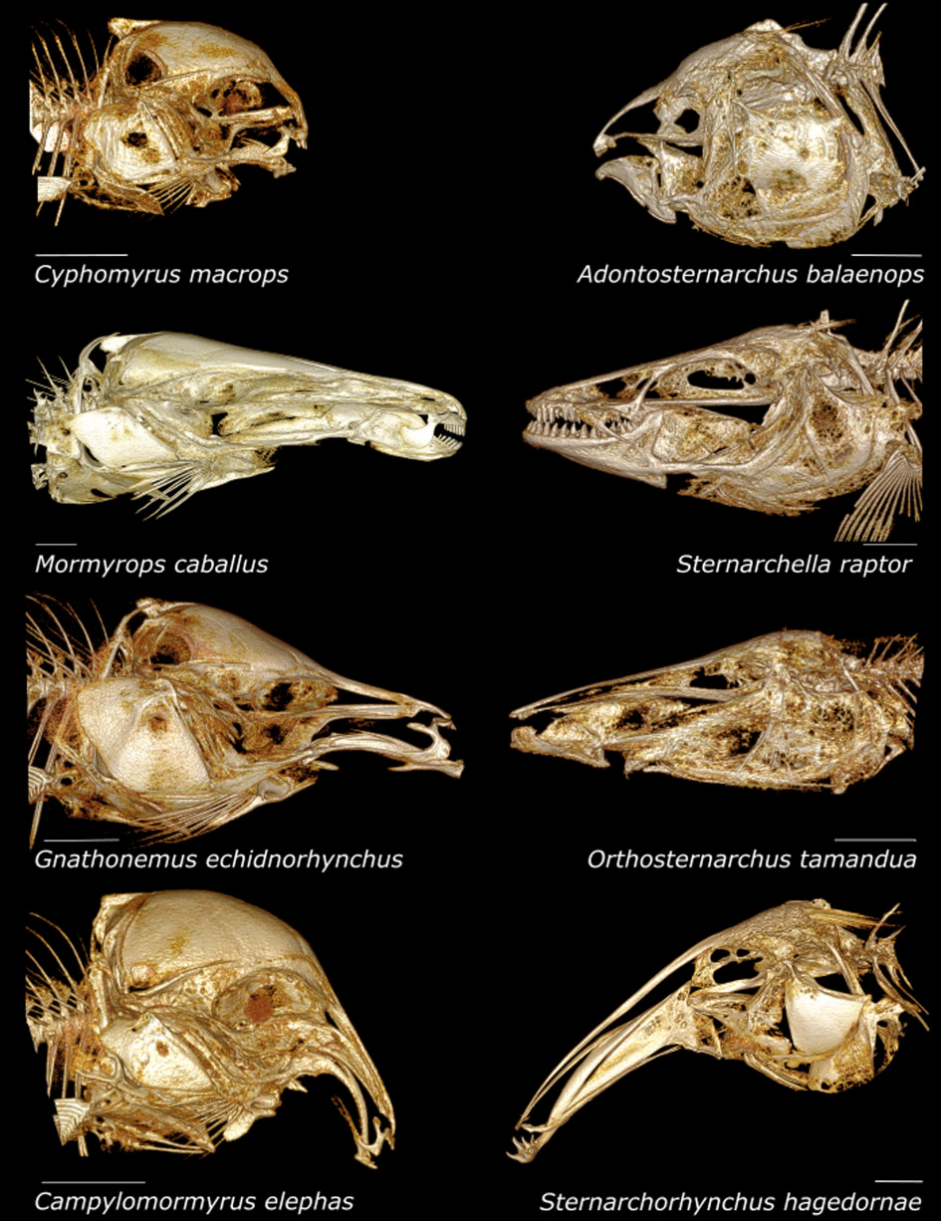
Skull images of four apteronotid and four mormyrid species. Four species each of Mormyridae (left) and Apteronotidae (right) showing diversity of skull shape among four morphotypes in lateral views. Each family includes species with a short snout and gracile oral jaws (top row), intermediate-length snout with robust oral jaws (second row), long straight snout (third row), and long decurved snout (bottom row). Scale bars for each image 5 mm.

Gymnotiformes and Mormyridae have long been viewed as a case of convergent evolution, including genetic, physiological, morphological, and behavioral traits associated with active electroreception (e.g., electrosensory receptor organs and central neural pathways, electrocytes and electromotor neural pathways), but also craniofacial phenotypes associated with trophic behaviors (Figs. 2 and 3) (Bullock and Heiligenberg 1986; Marrero and Winemiller 1993; Winemiller and Adite 1997; Zakon et al. 2006; Gallant et al. 2014). A large portion of the work on electric fish convergence has focused on similarities in electric signal (both electroreception and electrogeneration) and the genetic basis of signal diversity, and have found evidence for convergence in these areas (Bullock et al. 1983; Zakon et al. 2006; Lavoué et al. 2012; Wang and Yang 2021). In a few species of gymnotiform and mormyrid, there is evidence of convergence in external morphologies (Winemiller and Adite 1997), and qualitatively in osteological characters (Marrero and Winemiller 1993).

In our study, we quantitatively assessed similarities in craniofacial morphology across a diverse sampling of species in Apteronotidae and Mormyridae (Fig. 3), including 78 total species (40 of 227 mormyrid species, 36 of 97 apteronotid species, and two outgroups). We used 3D geometric morphometrics and phylogenetic comparative methods to determine if there is significant craniofacial similarity among species between these families. The aims of this study were to: (1) obtain a diverse open-source CT dataset for both families; (2) quantify craniofacial shape using 3D geometric morphometrics; and (3) statistically assess convergence across species of apteronotids and mormyrids hypothesized to be convergent using phylogenetic and morphological data.

## Materials and methods

### Specimen preparation

We scanned 391 specimens from two families of weakly electric fishes, the Apteronotidae (*n* = 162) and Mormyridae (*n* = 229), and reconstructed them for geometric morphometric analysis. Our species coverage was dense, with 78 species (an average of 5 specimens per species sampled); 40 of 227 mormyrid species and 36 of 97 apteronotid species (Supplemental Table 1) were included in this analysis. Apteronotids were caught in rivers near Iquitos, Peru in 2016–2017, and housed at the University of Louisiana at Lafayette. Mormyrids were borrowed from museum and academic collections (Cornell University Museum of Vertebrates and Texas A&M University-Corpus Christi). All specimens were aged as sub-adult or adult based on levels of ossification. The specimens were CT-scanned at Friday Harbor Labs with a Bruker SkyScan 1173 and the following parameters: 60–70kv and 114–133uA, and voxel sizes between 17.0–35.7μm. The scans were isolated using *CT-Vox* and *DataViewer*, and individual fish segmented using *3D-Slicer* (Fedorov et al. 2012). We generated surface renderings and volumes for geometric morphometrics (Fig. 3). We used one scan of the skull of outgroup species for each ingroup clade (Hiodontidae and Pimelodidae) deposited at MorphoSource.org (ark:/87602/m4/M51250; ark:/87602/m4/M53402). We deposited all our scan data at MorphoSource.org.

### Geometric morphometrics

**Fig. 4:**
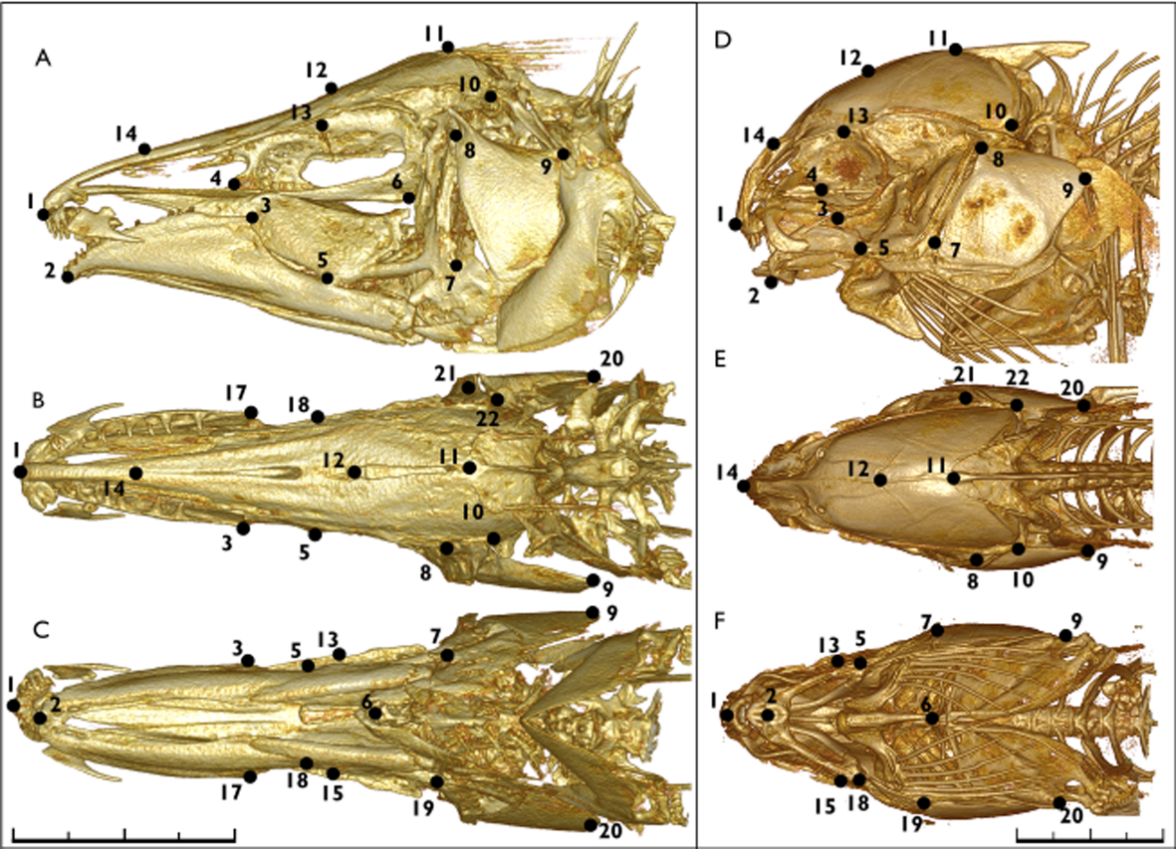
Landmark scheme for Apteronotidae and Mormyridae. Three-dimensional (3D) models of the head skeleton and pectoral girdle in the apteronotid *Apteronotus rostratus* (left) and the mormyrid *Ivindomyrus marchei* (right) with the 22-point landmark scheme used for geometric morphometric analyses. (a/d) Lateral (b/e) dorsal and (c/f) ventral views of the 3D model; anterior to left. See Table 1 for landmark definitions. Each scale bar is 10 mm.

We performed three-dimensional geometric morphometrics in *3D-Slicer* using a 22-point homologous landmark scheme across the entire skull (neurocranium, suspensorium, and lower jaw) (Fig. 4, Table 1) (Federov et al. 2012). We used the R package *geomorph* to complete a generalized Procrustes superimposition to remove the effects of size, rotation, and relative location from the shape analysis (Collyer and Adams 2018; Adams et al. 2021; RStudio Team).

**Table 1:** Landmark scheme for Apteronotidae and Mormyridae. Locations of homologous landmarks in the 22-point landmark scheme used in 3D geometric morphometrics analyses

LM #	Definition
1	Most anterior point of the mesethmoid (nasal septum)
2	Most anterior point of dentary
3	Most posterior point of dentary (L)
4	Articulation point between paraspenoid and prefrontal (L)
5	Articulation point between articular and quadrate (L)
6	Most posterior point of paraspenoid within jaw
7	Most posterior point of metapterygoid (L)
8	Articulation point between opercle and hyomandibular bone (L)
9	Most posterior point of opercle (L)
10	Most anterior point of post-temporal bone (L)
11	Most anterior point of supraoccipital crest
12	Articulation point between pariental and frontal bone
13	Articulation point between alispenoid and frontal (L)
14	Articulation point between frontal and pre-maxilla
15	Articulation point between alispenoid and frontal (R)
16	Articulation point between paraspenoid and prefrontal (R)
17	Most posterior point of dentary (R)
18	Articulation point between articular and quadrate (R)
19	Most posterior point of metapterygoid (R)
20	Most posterior point of opercle (R)
21	Articulation point between opercle and hyomandibular bone (R)
22	Most anterior point of post-temporal bone (R)

### Phylogenetic tree

A combined phylogenetic tree was generated using pruned versions of the Bernt et al. (2019) apteronotid phylogeny and the Peterson et al. (2022) mormyrid phylogeny (only species sampled in this study were kept in the new phylogeny). Bayesian and maximum-likelihood methods were used to infer each phylogeny based on multiple nuclear and mitochondrial genes (Bernt et al. 2019, Peterson et al. 2022). These two phylogenies were then combined in R (v.4.0.3) using the command *bindtree* in the R package *ape* (v. 5.4–1, Paradis and Schliep 2019). Next, the *chronos* function *Tree* (*ape)* and six fossil and secondary calibrations from Peterson et al. (2022) and Arcila and James 2017 were used to time-calibrate the combined topology (Paradis and Schliep 2019).

### Shape analysis

We completed a principal component analysis (PCA) in *geomorph* using all specimens to identify the major axes of shape change and visualize shape differences within and between families (Collyer and Adams 2018; Adams et al. 2021). A phylomorphospace was generated in *MorphoJ* using species shape averages to visualize phylogenetic relationships and identify potential instances of convergence (Klingenberg 2011). We completed an analysis of morphological disparity by genera which showed significant distances between multiple genera in our dataset (Supplemental Table 2).

### Assessment of convergence

We used two methods to test for convergence: the R packages *convevol* and *windex* (Arbuckle et al. 2014; Arbuckle 2015; Stayton 2015). The package *convevol* uses *a priori* hypotheses of convergence based on similarities in external phenotypes (Table 2) and assesses the phenotypic distance between taxa and the most recent common ancestor (Stayton 2015). Values of C1 describe the strength of convergence (distance between proposed tips divided by the maximum distance between any pair of taxa in those lineages) and C2 is the absolute amount of morphological evolution during convergence (subtract the maximum distance between two species from the distance between tips of proposed taxa) (Stayton 2015). We tested several groups of potentially convergent species based on proximity and phylogenetic distance in the phylomorphospace (based on species averages) and used groupings that provided the highest degrees of convergence. We also calculated the Wheatsheaf index (Arbuckle 2015) to test for convergence and assess the degree to which incomplete convergence best describes our data. This method also uses *a priori* hypotheses (Table 3) and calculated the ratio of the mean distances between all species to the distances between focal species (using species averages).

**Table 2:** Proposed groups of convergent apteronotid and mormyrid species for *convevol* analysis. Proposed convergence groups of apteronotid and mormyrid species based on qualitative proximity on the phylomorphospace. These groups were used in an analysis of convergence based on the distance between the hypothesized convergent taxa divided by the maximum distance between any two species in trait space. The results from the *convevol* analysis are included to show significant levels of convergence across some of the hypothesized groups

Species	Proposed Group
Sternarchorhynchus marreroi (A)	A
Campylomormyrus tamandua (M)	A
Mormyrops caballus (M)	A
Mormyrus proboscirostris (M)	A
Orthosternarchus tamandau (A)	B
Sternarchorhamphus muelleri (A)	B
Gnathonemus echidnorhynchus (M)	B
Pariosternarchus amazonensis (A)	C
Gnathonemus longibarbis (M)	C
Gnathonemus petersii (M)	C
Compsaraia sp (A)	D
Porotergus duende (A)	D
Hyperopisus bebe (M)	D
Adontosternarchus nebulosus (A)	E
Brevimyrus niger (M)	E
Petrocephalus catostoma (M)	E
Pollimyrus nigricans (M)	E

**Taible 3:** Proposed groups of convergent apteronotid and mormyrid species for *windex* analysis. Proposed convergence groups of apteronotid and mormyrid species based on qualitative proximity on the phylomorphospace. These groups were used in an analysis of convergence comparing the mean phenotypic distances between all species and the distances between our species of interest. The results from the *windex* analysis are included to show non-significant levels of convergence across all the hypothesized groups

Species	Proposed Group	Wheatsheaf Index	Lower 95%	Upper 95%	*P*-value
Sternarchorhynchus marreroi (A)	A	1.7952	1.7535	2.2182	0.435
Campylomormyrus tamandua (M)	A				
Mormyrops caballus (M)	A				
Mormyrus proboscirostris (M)	A				
Orthosternarchus tamandau (A)	B	2.095	2.017	2.474	0.749
Sternarchorhamphus muelleri (A)	B				
Gnathonemus echidnorhynchus (M)	B				
Pariosternarchus amazonensis (A)	C	0.912	0.890	1.026	0.539
Gnathonemus longibarbis (M)	C				
Gnathonemus petersii (M)	C				
Compsaraia sp (A)	D	2.243	2.188	2.954	0.313
Porotergus duende (A)	D				
Hyperopisus bebe (M)	D				
Adontosternarchus nebulosus (A)	E	1.442	1.400	1.577	0.666
Brevimyrus niger (M)	E				
Petrocephalus catostoma (M)	E				
Pollimyrus nigricans (M)	E				
Sternarchorhynchus marreroi (A)	A	1.899	1.838	2.269	0.271
Campylomormyrus tamandua (M)	A				
Mormyrops caballus (M)	A				
Mormyrus proboscirostris (M)	A				
Orthosternarchus tamandau (A)	B	1.245	1.204	1.465	0.839
Sternarchorhamphus muelleri (A)	B				
Gnathonemus echidnorhynchus (M)	B				
Pariosternarchus amazonensis (A)	C	1.198	1.160	1.412	0.344
Gnathonemus longibarbis (M)	C				
Gnathonemus petersii (M)	C				
Compsaraia sp (A)	D	2.924	2.829	3.476	0.206
Porotergus duende (A)	D				
Hyperopisus bebe (M)	D				
Adontosternarchus nebulosus (A)	E	1.629	1.576	2.079	0.538
Brevimyrus niger (M)	E				
Petrocephalus catostoma (M)	E				
Pollimyrus nigricans (M)	E				

## Results

### Morphological diversity

**Fig. 5:**
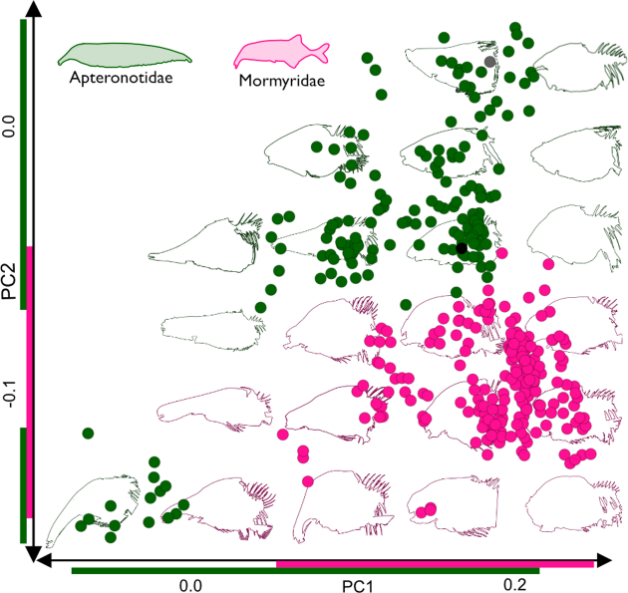
Principal Components Analysis for Apteronotidae and Mormyridae. A morphospace from a principal components analysis, with all sampled specimens. This morphospace shows skull shape disparity among 36 apteronotid (green circles) and 40 mormyrid (pink circles) species. Grey dot (*Aguarunichthys torosus*) and black dot (*Hiodon alosoides*) show the position of outgroups. Skull shape outlines in the background illustrate representative species for different areas of the morphospace. Note heterocephaly weights highly on PC1, and heterorhynchy on PC2. Note also apteronotid and mormyrid skulls do not overlap in this morphospace, although specimens come into close proximity at the center of the morphospace, and the absence of phenotypes in the top left corner of the morphospace (an elongate snout with upturned snout). Green and pink lines on the axes show the morphological coverage of each family.

There are high levels of diversity in craniofacial morphology across the two groups, and the PCA shows broad coverage of the morphospace by both families, with no overlap between them when PC1 and PC2 are the axes of shape change (Fig. 5). The first three PC axes represent 62.71% of the morphological variation for both Apteronotidae and Mormyridae (with outgroups), although PC3 (12.0%) does not show a singular phenotypic trend. PC1 (33.79%) represents the shape change trend *heterocephaly* (Evans et al. 2017), while PC2 (16.92%) visualizes the shape change trend *heterorhynchy* (Ford et al. 2022). Heterocephaly is the inverse relationship between the size of the snout (the pre-orbital portion of the skull) and braincase. Extreme PC1 phenotypes include *Sternarchorhynchus* and *Petrocephalus*. Heterorhynchy is the relative dorso-ventral flexion of the snout. These trends are not only seen when visualizing both families together but also in independent studies (Evans et al. 2017; Ford et al. 2022). Extreme morphologies of PC2 include *Sternarchella* and *Sternarchorhynchus*. There were significant differences in morphological disparity (based on the Procrustes absolute distances across genera) both within each family and across each family (Supplemental Table 2).

### Craniofacial evolution

**Fig. 6:**
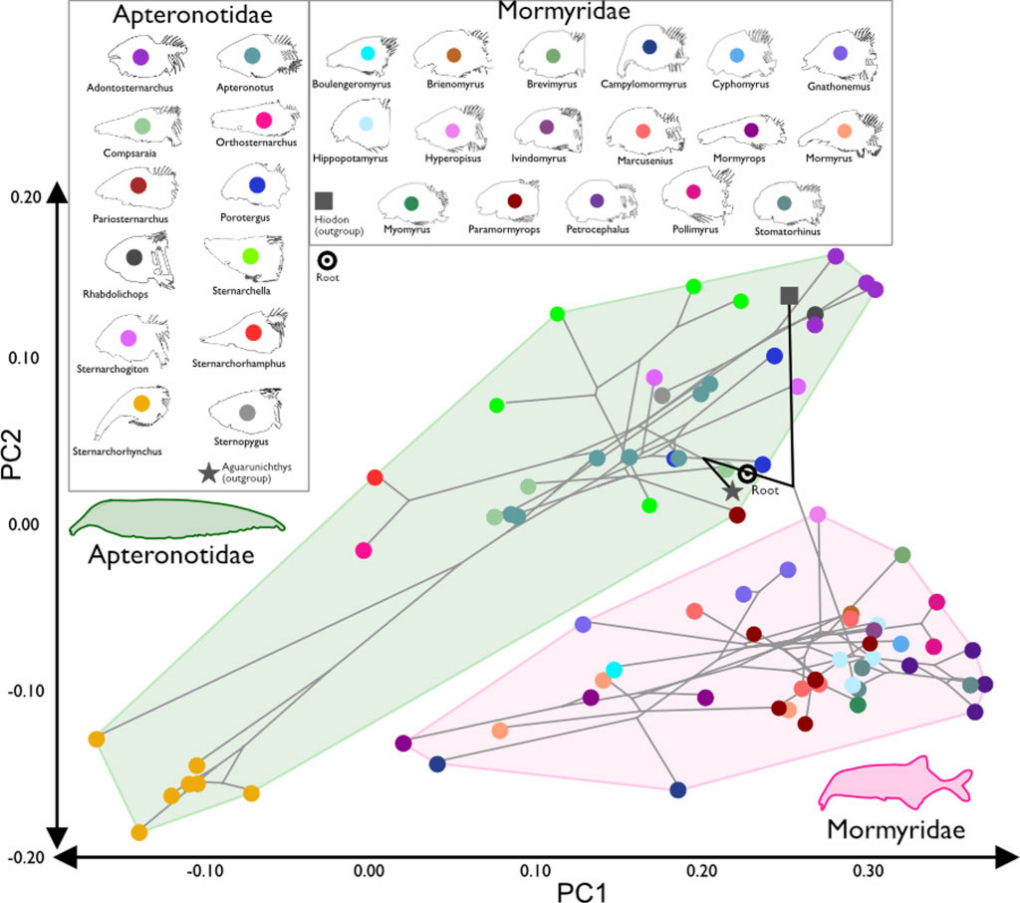
Phylomorphospace for Apteronotidae and Mormyridae. A phylomorphospace showing the phylogenetic relationships among taxa superimposed on the morphospace. Note there is no overlap between Apteronotidae and Mormyridae, and therefore no complete convergence of skull shape between taxa in these groups. Colored circles for each genus as in the insets.

**Fig. 7:**
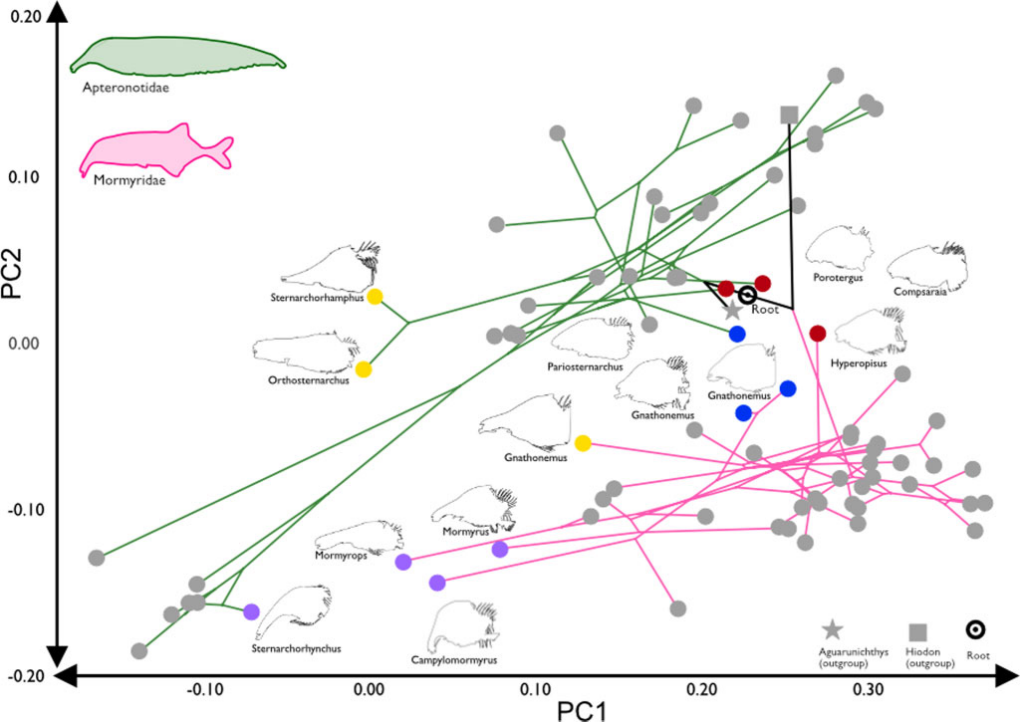
Convergence phylomorphospace for Apteronotidae and Mormyridae. The phylomorphospace showing groups of convergent taxa. Colored circles represent clades with partial skull convergence. Purple: *Sternarchorhynchus marreroi*, *Campylomormyrus tamandua*, *Mormyrops caballus*, *Mormyrus proboscirostris*. Yellow: Sternarchorhamphinae (*S. muelleri*, *O. tamandua*), *Gnathonemus echidnorhynchus*. Blue: *Pariosternarchus amazonensis*, *Gnathonemus petersii*, *Gnathonemus longibarbis*. Red: *Porotergus duende*, *Compsaraia samueli*, *Hyperopisus bebe*.

When both families are visualized in a phylomorphospace, there is no overlap between species averages of phenotypes (Fig. 6). There are instances of convergence and divergence within each family (Apteronotidae and Mormyridae), but no complete convergence between families. Instead, we see multiple instances of what we identify as incomplete convergence towards certain morphologies in each family and an additional parallel shift (Fig. 7; Simpson 1961; Schluter et al. 2004; Arendt and Reznick 2008; Bolnick et al. 2018). Based on the analyses in *convevol* (Table 2), there is an significant convergence on a dolichocephalic skull shape ([A] *Sternarchorhynchus marreroi*, *Campylomormyrus tamandua*, *Mormyrops caballus*, and *Mormyrus proboscirostris* where C1 = 0.71, *P* < 0.01) and two different convergences on intermediate-length snouts ([C] *Pariosternarchus amazonensis*, *Gnathonemus petersii*, and *Gnathonemus longibarbis* where C1 = 0.86, *P* < 0.01 and [D] *Porotergus duende*, *Hyperopisus bebe*, and *Compsaraia sp.*, where C1 = 0.80, p < 0.01). Based on the proximity in the morphospace, we classify this convergence as incomplete. The parallel shift is convergence between tube-snouted species ([B] *Sternarchorhamphus muelleri*, *Orthosternarchus tamandua*, and *Gnathonemus echidnorhynchus* where C1 = 0.67, *P* < 0.01), but where the taxa are remain physically separated on the morphospace but lines of the phylomorphospace show similar phenotypic shifts over time (similar slopes). Analyses of brachycephalic phenotypes [E] did not reveal convergence (C1 < 0.50). The results from the *windex* analysis, show non-significant Wheatsheaf indices in all our hypothesized groups of convergent species (Table 3). These results confirm our earlier result that there is no complete convergence between Apteronotidae and Mormyridae.

## Discussion

Many aspects of the phenotype in the electric fishes of the Afrotropics (Mormyroidea) and Neotropics (Gymnotiformes) have been interpreted as convergent, including: genes, cells and tissues of the electrosensory and electromotor systems, foraging and sexual behaviors, and foraging ecology (Bullock et al. 1983; Zakon 1986; Marrero and Winemiller 1993; Winemiller and Adite 1997; Zakon et al. 2006; Gallant et al. 2014). Researchers recognized similarities in external head and body morphology among distantly related taxa, hypothesizing that these aspects of external morphology reflect similar internal structures and functions; for example, grasp-suction feeding (Marrero and Winemiller 1993, Winemiller and Adite 1997). In our study of craniofacial evolution, however, we find a more complicated story of convergence, divergence, and independent trait evolution at different scales.

The two electric fish clades (Mormyridae and Apteronotidae) are completely separated on the PCA and phylomorphospace, with no instances of overlap in craniofacial morphology (Figs. 5 and 6). This is not what we expect if there were “complete” morphological convergence (*sensu*Losos 2011), and this interpretation is confirmed with the Wheatsheaf indices of potentially convergent taxa. In this sense convergence is superficial in that there have been multiple instances of incomplete morphological convergence, and one instance of a parallel shift (Fig. 7) (Stayton 2015; Grossnickle et al. 2020). This lack of complete convergence in head shape between mormyrid and apteronotid electric fishes is presumably based on developmental canalization (Evans et al. 2017), such that the convergent phenotypes exhibit a mosaic pattern of diversification and may be said to be only skin deep. Furthermore, the instances of incomplete convergence may be closely related to ecological factors such as diet preference and habitat occupancy. The extremely dolichocephalic mormyrid species (e.g., *Campylomormyrus*) has substrate preferences based on morphology (Amen 2020), with longer snouts allowing for moving substrate for foraging. Although it has not been examined in the same laboratory setting, the same is hypothesized regarding dolichocephalic apteronotid species.

Mormyrids and apteronotids occupy non-overlapping areas of the common skull morphospace, and mormyrids exhibit less total disparity (Figs. 5 and 6). Both families include species with foreshortened and elongate skulls, but some apteronotids (i.e., *Sternarchorhynchus*) exhibit the most extreme dolichocephalic phenotypes in the morphospace (Fig. 5). Mormyrids have shorter skulls in general, and *Petrocephalus* occupies the most extreme brachycephalic portion of the morphospace. The mormyrids sampled do not occupy the extreme dolichocephalic (long snouts with a small braincase) end of the continuum in part because they have a relatively larger brain and braincase than do apteronotids (Fig. 5; Carlson et al. 2011; Carlson and Gallant 2013; Stevens et al. 2013).

**Fig. 8:**
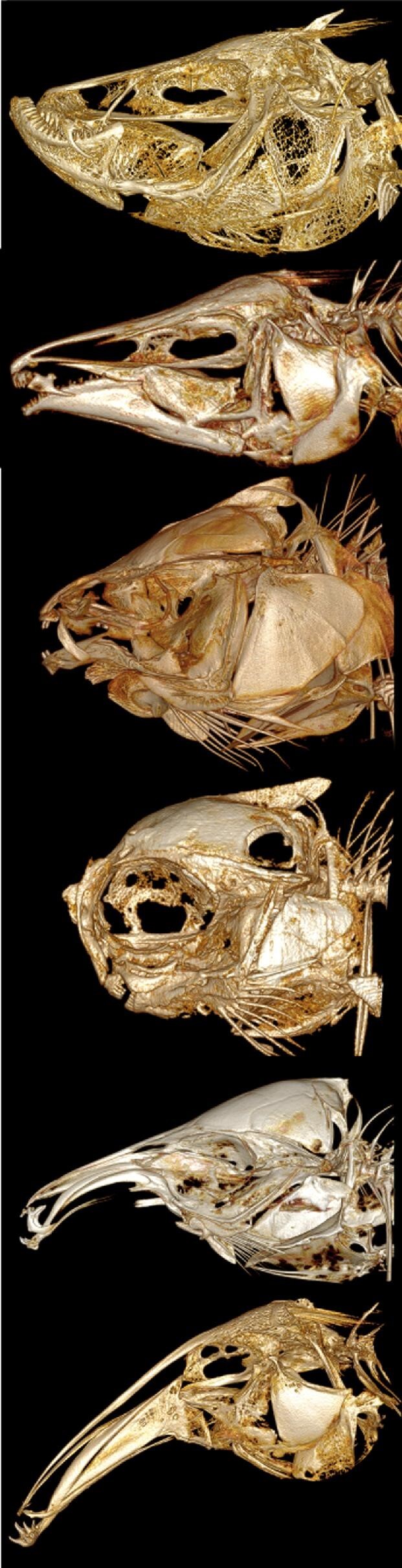
Diversity of skull phenotypes in Apteronotidae and Mormyridae. Five species depicting the diversity in heterorhynchy phenotypes: *Sternarchella duccis* (A), *Apteronotus rostratus* (A), *Hyperopisus bebe* (M), *Petrocephalus grandoculis* (M), *Campylomormyrus tamandua* (M), and *Sternarchorhynchus hagedornae* (A). Apteronotids have representatives with concave and convex heterorhynchy, along with terminal mouths. Mormyrids have either convex heterorhynchy or terminal mouths.

Mormyrids also occupy the exact range of PC2 values left unoccupied by apteronotids in the PCA, indicating stark differences in mouth position and dorso-ventral flexion of apteronotid and mormyrid snouts (Fig. 5). Mormyrids may be morphologically constrained by sensory related tissues that extend into the nasal region, leading to sub-terminal mouths and rounded foreheads (Carlson et al. 2011; Carlson and Gallant 2013; Stevens et al. 2013). An extreme example is *Petrocephalus* with very large bony canals at the anteriormost region of the skull (Fig. 8). In contrast, many apteronotids have terminal mouths, perhaps because some species have a habit of male combat which involves biting the tail of competitors (Lundberg et al. 1996; Albert and Crampton 2009). A terminal mouth in the apteronotid taxa that have it may therefore allow the ethmoid region of the skull more dorso-ventral freedom to flex forming concave-down or convex-up snout morphologies (Fig. 8).

Although weakly electric fishes occupy a large portion of the morphospace, there is a large, empty area with no representatives of either group (low PC1 values coupled with high PC2 values). This empty region of the morphospace is where we would see species with dolichocephalic, upturned snouts, a phenotype not observed in apteronotids or mormyrids (Ford et al. 2022, Ford et al. 2022), but which is observed in some other teleost fishes (Aulostomidae, Syngnathidae, etc.) (de Lussanet and Muller 2007; Lees et al. 2012). We interpret this empty region of the morphospace as a constraint in apteronotids and mormyrids, although not among teleost fishes in general. The relatively distant evolutionary relationship of apteronotids and mormyrids, with a most recent common ancestor approximately 150 million years ago, suggests that shared history is a poor explanation for the “missing phenotypes” in these clades. The role of genetic or developmental mechanisms underlying these phenotypic constraints could be explored using genetic editing methods and ontogenetic studies across multiple taxa.

## Conclusion

The story of craniofacial evolution between Afrotropical mormyrid and Neotropical apteronotid electric fishes is complex, illustrating several common themes in comparative biology. The morphological similarities observed within each clade exhibit a mosaic pattern of occurrence among species, with many examples of phylogenetic convergence, divergence and stasis (conservatism). Individual traits may be convergent at one or more levels in the hierarchy of biological organization (e.g., cellular, tissue, organ, whole body) and not necessarily at other levels. Certain phenotypes of external anatomy (e.g., body size, head and mouth shape, eye size, fin configuration, etc.) may be similar despite different underlying structures (e.g., skeletal, musculature, nervous innervation, etc.). Although these external phenotypes evolved to perform certain functions and behaviors, they are not necessarily built by ontogenies in the same way. In this sense, morphological convergence between these groups may be viewed as superficial.

Supplemental Table 1. Catalog Information. Catalog and collection information for apteronotid and mormyrid species sampled in this analysis. Specimen numbers, museum ID numbers, and *n*-numbers are included.

## Supplementary Material

obac022_Supplemental_File

## Data accessibility

CT scans of all individuals are being made freely-available through the open-source web program MorphoSource.
